# Increased suicide‐related behaviors, challenging behaviors, and anxiety symptoms in elementary and junior high school students after the coronavirus disease 2019 pandemic: A single‐center case–control study

**DOI:** 10.1002/pcn5.70263

**Published:** 2025-12-02

**Authors:** Masahiro Ishida, Yoshinori Sasaki, Masahide Usami, Yuta Yoshimura, Masaya Ito, Katsunaka Mikami, Noa Tsujii, Hiroaki Kihara, Yuriko Yanagi, Tamae Inomata, Kotoe Itagaki, Ayaka Hashimoto, Keita Yamamoto, Yuki Hakoshima, Kumi Inazaki, Yuki Mizumoto, Hikaru Hori

**Affiliations:** ^1^ Department of Psychiatry, Faculty of Medicine Fukuoka University Fukuoka Japan; ^2^ Department of Child and Adolescent Psychiatry, National Kohnodai Medical Center Japan Institute for Health Security Chiba Japan; ^3^ Department of Psychiatry and Behavioral Sciences Institute of Science Tokyo Graduate School of Medical and Dental Sciences Tokyo Japan; ^4^ National Center for Cognitive Behavior Therapy and Research National Center of Neurology and Psychiatry Kodaira Japan; ^5^ Department of Psychiatry Tokai University School of Medicine Kanagawa Japan; ^6^ Department of Child Mental Health and Development Toyama University Hospital Toyama Japan; ^7^ Department of Neuropsychiatry Kanazawa Medical University Ishikawa Uchinada Japan; ^8^ Department of Psychiatry, National Kohnodai Medical Center Japan Institute for Health Security Chiba Japan

**Keywords:** child and adolescent psychiatry, COVID‐19, developmental differences, pandemic, suicide‐related behavior

## Abstract

**Aim:**

The coronavirus disease 2019 (COVID‐19) pandemic has had a significant psychological impact on children and adolescents, increasing depression, anxiety, and suicide‐related behaviors. In Japan, suicide remained the leading cause of death among individuals aged 10–19 years, with rates rising after the pandemic onset. However, few studies have examined these changes in psychiatric outpatient settings. We aimed to determine whether the prevalence of suicide‐related behaviors and associated psychiatric symptoms—depression and anxiety—differed pre‐ and post‐pandemic among elementary and junior high school students attending psychiatric outpatient clinics.

**Methods:**

In this retrospective case**–**control study, we analyzed registry data from a child and adolescent psychiatric outpatient clinic in Japan. Patients were classified into pre‐ and post‐COVID‐19 groups based on the visit date (cutoff: March 2, 2020). Clinical characteristics were assessed at the initial visit through interviews and standardized rating scales: Depression Self‐Rating Scale for Children, Spence Children's Anxiety Scale, and Attention‐Deficit/Hyperactivity Disorder Rating Scale‐IV. Group differences were examined using univariate and multivariate logistic regression analyses.

**Results:**

Between 2016 and 2022, 2878 patients were included. The prevalence of suicide‐related behaviors increased post‐pandemic, from 3.0% to 6.9% among elementary school students and from 15.5% to 21.2% among junior high school students. In the post‐COVID‐19 group, elementary students more often exhibited antisocial behaviors and hyperactivity/conduct disorder diagnoses. Junior high students more often exhibited anxiety symptoms, particularly social anxiety, panic, and trauma‐related fear.

**Conclusion:**

Suicide‐related behaviors significantly increased after the COVID‐19 pandemic, with distinct clinical characteristics observed across age groups.

## INTRODUCTION

Coronavirus disease 2019 (COVID‐19) emerged in 2019 and rapidly spread worldwide. In several countries, strict social distancing was strongly recommended to limit transmission. However, mandatory social distancing had various adverse effects on mental health. Individuals potentially exposed to the virus reported psychological symptoms such as post‐traumatic stress disorder, anxiety, irritability, and depression, largely due to movement restrictions and isolation.^1^ Access to mental health services was also limited, as infection control took precedence.^2^ Long‐term school closures in many countries disproportionately affected young people, increasing the risk of suicide attempts, suicidal ideation, anxiety, and depression.^3^ A meta‐analysis reported that the prevalence of depressive symptoms among children and adolescents rose from 12.9% post‐pandemic to 25.2% during the pandemic, while anxiety symptoms rose from 11.6% to 20.5%.^4^ However, findings on anxiety symptoms have been inconsistent, with outcomes influenced by age, sex, socioeconomic status, and family functioning.^5^ A systematic review of suicide‐related behaviors after COVID‐19 onset reported increases in suicidal ideation, suicide attempts, and self‐harm, with young people and women identified as vulnerable populations.^6^

In Japan, consistent with global trends, an increase in depressive symptoms among young people and their caregivers was reported after the pandemic.^7^ Japan remains the only G7 country where suicide is the leading cause of death among individuals aged 10–19.^8^ Since the pandemic onset, suicide among elementary to high school students has continued to increase, reaching a record high of 527 in 2024.^9^ A closer analysis of this sharp increase since 2020 revealed differences by sex and school level, with suicides rising among high school boys and both junior high and high school girls.^9^ According to the Ministry of Health, Labor and Welfare, the most frequently reported reason for suicide among female junior high school students was “unknown,” precluding the identification of specific causes. A demographic study of suicides among Japanese youth aged 10–19 reported an increase in suicide‐related behaviors after the COVID‐19 outbreak, identifying family problems, limited access to mental health services, and stress‐related exacerbation of psychiatric symptoms as possible contributing factors.^10^ However, because that study lacked clinical assessments of psychiatric symptoms, its findings provided a limited understanding.

To our knowledge, few studies have simultaneously evaluated suicide‐related behaviors and psychiatric symptoms using both self‐report questionnaires and clinical assessments conducted by child and adolescent psychiatrists and psychologists. Anxiety disorders and depression have been associated with suicide‐related behaviors in adolescents aged 12–17 years.^11^ However, the onset of psychiatric symptoms during adolescence varies by age,^12^ highlighting the need for more detailed subgroup analyses.

Previous studies have also documented increases in suicide‐related behaviors among adolescents following the onset of the COVID‐19 pandemic.^10^, ^11^ However, the populations and settings examined in these studies varied considerably. For instance, Goto et al. analyzed nationwide suicide statistics among Japanese youth aged 10–19 years, focusing on overall demographic trends without clinical assessment of psychiatric symptoms.^10^ In contrast, Gracia‐Liso et al. examined adolescents aged 12–17 years who presented to psychiatric emergency services in Spain for suicide attempts, identifying diagnostic shifts before and after the pandemic.^11^ The present study extends this evidence by examining a younger outpatient population—elementary and junior high school students—who sought initial psychiatric consultation for various emotional and behavioral problems. This outpatient‐based design allowed exploration of developmental differences and detailed psychiatric symptom profiles not captured in large‐scale epidemiological or emergency‐based studies.

In this study, we aimed to examine changes in suicide‐related behaviors and psychiatric symptoms—particularly depression and anxiety—among elementary and junior high school students before and after the COVID‐19 pandemic, using registry data from a child and adolescent psychiatric outpatient clinic.

Our primary objective was to determine whether the proportion of elementary and junior high school students presenting with suicide‐related behaviors at a psychiatric outpatient clinic increased after the COVID‐19 pandemic compared with the pre‐pandemic period. We hypothesized that this proportion would be higher in the post‐pandemic period. In addition, we explored whether depressive and anxiety symptom scores differed between the pre‐ and post‐pandemic periods among outpatient elementary and junior high school students. To address these aims, we employed a retrospective case–control design. Considering developmental differences, we first analyzed the overall sample and then conducted exploratory subgroup comparisons between elementary and junior high school students.

## METHODS

### Study design and setting

The Department of Child and Adolescent Psychiatry at the National Kohnodai Medical Center, part of the Japan Institute for Health Security, began collecting data on first‐time outpatient visits in January 2016, before the COVID‐19 pandemic. This dataset enabled a consistent comparison of the patient characteristics before and after the pandemic using the same assessment tools.

This study was a retrospective case**–**control analysis using registry data. We examined whether suicide‐related behaviors among elementary and junior high school students increased after the onset of the COVID‐19 pandemic and whether psychiatric symptoms among students with suicide‐related behaviors differed between the two periods.

We identified all first‐time patients who visited the clinic between January 1, 2016, and December 31, 2022. Duplicate entries were removed, and only the most recent record for each patient was retained, as this best reflected clinical status for evaluating changes associated with the COVID‑19 pandemic. Based on reports that school closures can influence young people's mental health,^13^, ^14^ we set March 2, 2020, as the cutoff date. On February 27, 2020, the Japanese government requested a nationwide temporary school closure, and March 2 was the first day this policy was implemented.^15^ Students who first visited on or after that date were classified as the post‑COVID‑19 (case) group, while those who visited before were classified as the pre‑COVID‑19 (control) group. We first conducted a case–control analysis comparing all patients in the pre‑ and post‑COVID‑19 periods. Next, we extracted students with suicide‑related behaviors from both groups and conducted a second case–control analysis focused on these subgroups. Because each analysis was performed separately for elementary and junior high school students, we carried out four case–control comparisons in total.

### Recruitment of participants

Participants were patients who visited the Department of Child and Adolescent Psychiatry at the National Kohnodai Medical Center, Japan Institute for Health Security, for the first time between January 2016 and December 2022. The department accepts patients up to the ninth grade. All diagnoses were made by child and adolescent psychiatrists according to the International Classification of Diseases, 10th Revision (ICD‐10). Patients with moderate to severe intellectual disability, organic brain disease, substance‑induced psychotic disorders, traumatic brain injury, or genetic syndromes were referred to other hospitals at the initial appointment because they required specialized treatment; therefore, they were not included in the analyses. In addition, patients who were not enrolled in school at the time of assessment were excluded, as the study targeted elementary and junior high school students.

Among eligible patients, we initially identified 3119 first‐time outpatients. After excluding duplicates and retaining only the most recent record for patients with multiple entries, a total of 2878 patients were included in the final analysis.

### Procedure and assessment

At the initial visit, a psychiatrist and clinical psychologist interviewed the child and their caregiver to assess demographic characteristics, psychiatric symptoms (e.g., depression, anxiety), and suicide‐related behaviors. Diagnoses were made according to the ICD‐10. Suicide‐related behaviors that occurred before the initial visit were evaluated by the psychiatrist using clinical judgment, with reference to the Columbia Classification Algorithm of Suicide Assessment (C‐CASA),^16^ and categorized by method (e.g., drug overdose, wrist cutting, jumping from a height, and hanging). Standardized psychological rating scales were also administered to assess psychiatric symptoms: the Depression Self‐Rating Scale for Children (DSRS‐C), Spence Children's Anxiety Scale (SCAS), and Attention‐Deficit/Hyperactivity Disorder Rating Scale‐IV (ADHD‐RS‐IV).

DSRS‐C: This 18‐item self‐report questionnaire assesses depressive symptoms in children. Each item is rated on a 3‐point scale (0–2), yielding a total score ranging from 0 to 36; higher scores indicate more severe symptoms.^17^ The Japanese version was developed by Murata based on the original English scale.^18^ In their study of Japanese children aged 7–13 years, a cutoff score of 16 was proposed to distinguish between depressed and non‐depressed groups.^18^

SCAS: This 38‐item questionnaire assesses symptoms across several anxiety disorders, including social phobia, obsessive–compulsive disorder, and panic disorder. It can be completed by either the child or a guardian. Each item is rated on a 4‐point scale (0–3), with a maximum score of 114 suggesting the most severe anxiety symptoms.^19^ No established cutoff exists; however, previous studies have suggested a score of 42 indicating clinically significant anxiety.^20^ The validity and reliability of the Japanese version were confirmed by Ishikawa et al.^21^

ADHD‐RS‑IV: The ADHD‐RS‐IV was developed based on the diagnostic criteria of the Diagnostic and Statistical Manual of Mental Disorders, Fourth Edition. This 18‐item, caregiver‐completed scale assesses symptoms of ADHD. Each item is scored on a 4‐point scale (0–3), with total scores ranging from 0 to 54; higher scores indicate more severe symptoms.^22^ The standardized Japanese version includes two subscales: hyperactivity/impulsivity and inattention.^23^ Its reliability and validity have been confirmed in a large school‐age sample.^24^

### Ethical considerations

This study was approved by the Ethics Committee of the National Center for Global Medical Research (approval numbers NCGM‐G‐003042‐08 and NCGM‐S‐004487‐03) and conducted in accordance with the Declaration of Helsinki. In Japan, based on the “*Ethical Guidelines for Medical and Health Research Involving Human Subjects*,” personal information may be provided to third parties without explicit consent if individual consent is obtained and the individuals are given prior notice and an opportunity to refuse participation. In this study, prior notice was posted, and no written or verbal informed consent was obtained. The guidelines state, “It is not always necessary to obtain informed consent from research participants. However, for observational research using historical clinical records rather than human tissue samples, researchers must disclose information about the conduct of the research, including the purpose of the research.” Accordingly, details on the study's purpose, methods, analysis, and refusal options were posted in the hospital's outpatient clinic and on its website. During the study period, all data were anonymized to protect patient confidentiality.

### Statistical analysis

#### Participants and comparison of characteristics

For univariate analyses, we used chi‐square tests for categorical variables and the Mann–Whitney *U* test for continuous variables to compare clinical characteristics between the pre‐COVID‐19 (control group) and post‐COVID‐19 (case group) groups separately for elementary and junior high school students. For multivariate analyses, we conducted logistic regression with “pre‐ or post‐COVID‐19” as the dependent variable. Explanatory variables included those with statistically significant differences in univariate analyses or those deemed clinically relevant to suicide‐related behaviors.

#### Comparison of suicide‐related behaviors before and after the COVID‐19 pandemic

We compared the prevalence of suicide‐related behaviors pre‐ and post‐COVID‐19 pandemic separately for elementary and junior high school students. Chi‐square tests were used to examine whether the prevalence differed significantly between the groups. The primary outcome of this study was to determine whether the frequency of suicide‐related behaviors increased after the onset of the COVID‐19 pandemic.

#### Characteristics of students with suicide‐related behaviors before and after the COVID‐19 pandemic

To compare the characteristics of elementary and junior high school students with suicide‐related behaviors before and after the pandemic, we conducted separate analyses for the control and case groups. Chi‐square tests were used for nominal variables, and the Mann–Whitney *U* test was applied to continuous variables. For multivariate analyses, we performed logistic regression using “pre‐ or post‐COVID‐19” as the dependent variable. Explanatory variables were selected based on statistically significant differences in univariate analyses or their relevance to suicide‑related behaviors. Based on prior research^11^ and clinical experience, we included SCAS subscales as explanatory variables. Multicollinearity was assessed using the variance inflation factor (VIF), with VIF ≤ 5 considered acceptable. Multivariate analysis was not conducted for elementary school students with suicide‐related behaviors due to the small sample size. A *p* value < 0.05 was considered statistically significant. All statistical analyses were performed using Easy R version 1.67.^25^

## RESULTS

### Participants

**Figure 1:**
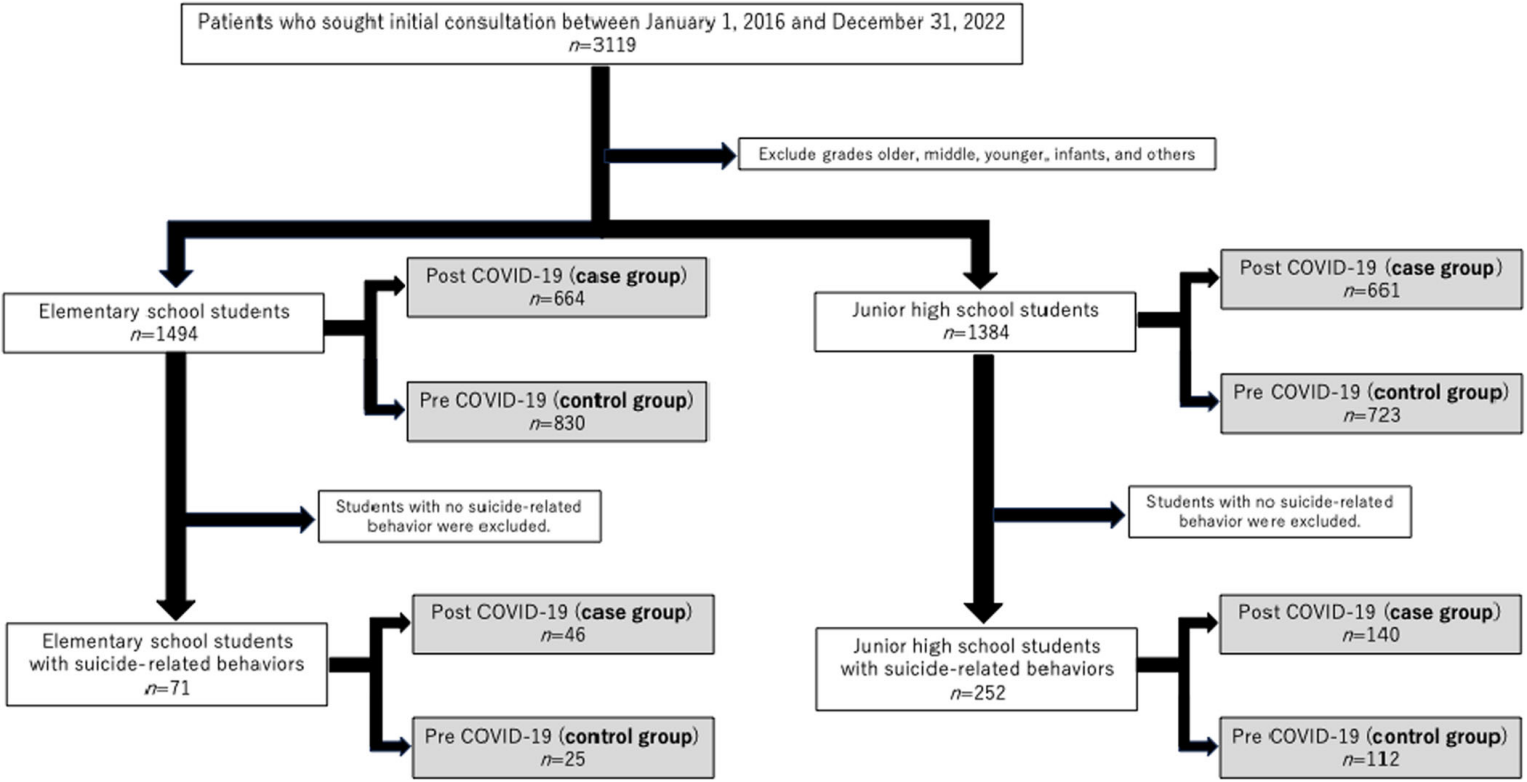
Flowchart of participant selection for case and control groups.

In total, 2878 individual patients visited the Department of Child and Adolescent Psychiatry at the National Kohnodai Medical Center, part of the Japan Institute for Health Security, between January 2016 and December 2022. Of these, 1494 were elementary school students, and 1384 were junior high school students. The mean age of participants was 11.2 years (standard deviation = 2.5), and 1286 (44.7%) were female. Participants were classified into two groups based on the timing of their first visit. The post‐COVID‐19 group (case group) included those who first visited after March 2, 2020 (the date of nationwide school closures due to the COVID‐19 pandemic). The pre‐COVID‐19 group (control group) included those who first visited before this date. Figure 1 provides an overview of the participant selection process.

### Suicide‐related behaviors before and after the COVID‐19 pandemic

Among elementary school students, the proportion of those with suicide‐related behaviors was 3.0% (*n* = 25) in the pre‐COVID‐19 group and 6.9% (*n* = 46) in the post‐COVID‐19 group. Among junior high school students, the proportions were 15.5% (*n* = 112) and 21.2% (*n* = 140), respectively. These differences were statistically significant in both groups (*p* < 0.01).

### Clinical characteristics by school age

#### Elementary school students

We analyzed the clinical characteristics of elementary school students pre‐ and post‐COVID‐19 pandemic. In the univariate analysis, suicide‐related behaviors and F4 diagnoses were significantly more common in the post‐COVID‐19 group (both *p* < 0.01). In contrast, antisocial behaviors and F9 diagnoses were more frequent in the pre‐COVID‐19 group (both *p* < 0.05). We conducted logistic regression with “pre‐ or post‐COVID‐19” as the dependent variable. Explanatory variables included those with statistically significant differences in univariate analyses or those deemed clinically relevant to suicide‐related behaviors. No variables were significantly associated with the timing of the pandemic in the multivariate model. Table 1 shows the results of univariate analyses for clinical variables in elementary school students with suicide‐related behaviors. Antisocial behaviors and F9 diagnoses were more frequently observed in the post‐COVID‐19 group (both *p* < 0.05). Multivariate analysis was not conducted for elementary school students with suicide‐related behaviors due to the small sample size.

**Table 1:** Comparison of clinical characteristics in elementary school students with suicide‐related behaviors before and after the COVID‐19 pandemic (*N* = 71).

Characteristics	Case (*N* = 46)	Control (*N* = 25)	OR	95% CI	*p* value
	% (*n*)	% (*n*)			
Female	39.1 (18)	60.0 (15)	0.43	0.14–1.29	0.14
Antisocial behavior	41.3 (19)	16.0 (4)	3.63	0.99–16.9	**<0.05**
Abuse history	23.9 (11)	20.0 (5)	1.25	0.34–5.28	0.77
F3	10.9 (5)	16.0 (4)	0.64	0.12–3.61	0.71
F4	32.6 (15)	40.0 (10)	0.73	0.24–2.28	0.61
F5	4.3 (2)	0 (0)	‐	‐	0.54*
F8	28.3 (13)	36.0 (9)	0.70	0.22–2.29	0.59
F9	17.4 (8)	0 (0)	‐	‐	**<0.05** *

Characteristics	*m* ± SD^a^ (*n*)	*m* ± SD^a^ (*n*)	Cohen's *d*		*p* value
Age	9.96 ± 1.81 (46)	10.36 ± 1.78 (25)	0.222		0.26
DSRS total^b^	17.49 ± 8.20 (41)	19.25 ± 7.09 (20)	0.224		0.4
SCAS total^c^	43.72 ± 25.66 (39)	37.24 ± 23.13 (17)	0.260		0.46
SCAS—separation anxiety	7.38 ± 4.63 (39)	6.29 ± 5.03 (17)	0.229		0.34
SCAS—social anxiety	7.31 ± 5.83 (39)	7.76 ± 4.41 (17)	0.083		0.43
SCAS—OCD	7.13 ± 4.27 (39)	6.12 ± 3.87 (17)	0.243		0.41
SCAS—panic	7.21 ± 7.36 (39)	5.47 ± 5.28 (17)	0.255		0.68
SCAS—generalized anxiety	6.82 ± 3.98 (39)	4.82 ± 3.78 (17)	0.510		0.1
SCAS—fear of trauma	7.87 ± 5.19 (39)	6.76 ± 3.96 (17)	0.228		0.62
ADHD‐RS^d^	21.12 ± 12.36 (41)	15.88 ± 13.14 (24)	0.414		0.09
ADHD‐RS—hyperactivity impulsivity	8.73 ± 7.00 (41)	6.50 ± 6.19 (24)	0.332		0.17
ADHD‐RS—inattention	12.39 ± 6.28 (41)	9.38 ± 7.91 (24)	0.441		0.07

*Note*: Statistically significant values are shown in boldface.

Abbreviations: ADHD‐RS, Attention‐Deficit/Hyperactivity Disorder Rating Scale; CI, confidence interval; DSRS, Depression Self‐Rating Scale; F3, mood disorders; F4, neurotic, stress‐related, and somatoform disorders; F5, behavioral syndromes associated with physiological disturbances and physical factors; F8, disorders of psychological development (e.g., autism spectrum disorder); F9, behavioral and emotional disorders with onset usually occurring in childhood and adolescence (e.g., ADHD); OCD, obsessive‐compulsive disorder; OR, odds ratio; SCAS, Spence Children's Anxiety Scale.

^a^ Mean ± standard deviation.

^b^ Depression Self‐Rating Scale for Children: 0–36 points.

^c^ Spence Children's Anxiety Scale: 0–114 points.

^d^ ADHD‐Rating Scale: 0–54 points.

* OR and 95% CI could not be estimated due to zero count in one of the groups.

#### Junior high school students

We analyzed the clinical characteristics of junior high school students pre‐ and post‐COVID‐19 pandemic. The post‐COVID‐19 group showed significantly higher proportions of females, students with suicide‐related behaviors, F5 diagnoses, and SCAS total scores (all *p* < 0.01), as well as F4 diagnoses and SCAS subscale scores for separation anxiety, social anxiety, panic, generalized anxiety, and fear of trauma (all *p* < 0.05) than the pre‐COVID‐19 group. In contrast, antisocial behavior, history of abuse, and ICD‐10 categories F8 (disorders of psychological development) and F9 were more prevalent in the pre‐COVID‐19 group (*p* < 0.01). ADHD‐RS scores, including total, hyperactivity/impulsivity, and inattention subscales, were also significantly higher in the pre‐COVID‐19 group than in the post‐COVID‐19 group (*p* < 0.05).

Table 2 presents the results of the multivariate logistic regression analysis examining differences in clinical characteristics between the pre‐ and post‐COVID‐19 groups. SCAS social anxiety scores were significantly higher in the post‐COVID‐19 group than in the pre‐COVID‐19 group (odds ratio [OR] = 1.08, *p* < 0.01). In contrast, female sex (OR = 0.61, *p* < 0.01) and DSRS total scores (OR = 0.97, *p* < 0.05) were significantly lower in the post‐COVID‐19 group. In this model, the maximum VIF was 4.15, below the threshold of 5, indicating minimal multicollinearity.

**Table 2:** Multivariate logistic regression analysis of clinical characteristics in junior high school students before and after the COVID‐19 pandemic (*N* = 1384).

Characteristics	OR	95% CI	*p* value	VIF
Female	0.61	0.48–0.79	**<0.01**	1.17
Age	1.09	0.95–1.25	0.22	1.03
DSRS total	0.97	0.95–1.00^a^	**<0.05**	2.25
SCAS separation anxiety	0.97	0.93–1.01	0.17	2.39
SCAS generalized anxiety	1.01	0.97–1.06	0.56	1.49
SCAS social anxiety	1.08	1.04–1.12	**<0.01**	2.84
SCAS OCD	0.99	0.95–1.03	0.51	2.01
SCAS fear of trauma	0.98	0.94–1.03	0.45	4.15
SCAS panic	1.02	0.98–1.05	0.37	3.56

*Note*: Statistically significant values are shown in boldface.

Abbreviations: CI, confidence interval; DSRS, Depression Self‐Rating Scale; OCD, obsessive‐compulsive disorder; OR, odds ratio; SCAS, Spence Children's Anxiety Scale; VIF, variance inflation factor.

^a^ The value was increased to 1.00 after rounding up to the third decimal place.

We analyzed the clinical characteristics of junior high school students with suicide‐related behaviors pre‐ and post‐COVID‐19 pandemic. In the univariate analysis, SCAS social anxiety scores were significantly higher in the post‐COVID‐19 group than in the pre‐COVID‐19 group (*p* < 0.01). Moreover, the proportion of females, SCAS total scores, and SCAS panic subscales were higher in the post‐COVID‐19 group than in the pre‐COVID‐19 group, while F8 was more prevalent in the pre‐COVID‐19 group (all *p* < 0.05).

Table 3 presents the results of the multivariate logistic regression analysis comparing clinical characteristics between the groups. SCAS social anxiety and panic scores were significantly higher in the post‐COVID‐19 group than in the pre‐COVID‐19 group (both *p* < 0.05), while SCAS fear of trauma scores were significantly lower in the post‐COVID‐19 group (*p* < 0.01). In the model restricted to junior high school students with suicide‑related behaviors, the maximum VIF was 4.39, below the threshold of 5, indicating minimal multicollinearity.

**Table 3:** Multivariate logistic regression analysis of clinical characteristics in junior high school students with suicide‐related behaviors before and after the COVID‐19 pandemic (*N* = 252).

Characteristics	OR	95% CI	*p* value	VIF
Female	0.63	0.31–1.28	0.20	1.20
Age	1.02	0.73–1.42	0.92	1.06
DSRS total	0.98	0.92–1.04	0.55	2.57
SCAS separation anxiety	0.99	0.90–1.09	0.89	2.01
SCAS generalized anxiety	0.98	0.89–1.09	0.73	1.58
SCAS social anxiety	1.10	1.01–1.19	**<0.05**	2.24
SCAS OCD	1.04	0.95–1.14	0.42	1.97
SCAS fear of trauma	0.86	0.76–0.96	**<0.01**	4.39
SCAS panic	1.08	1.01–1.17	**<0.05**	4.04

*Note*: Statistically significant values are shown in boldface.

Abbreviations: CI, confidence interval; DSRS, Depression Self‐Rating Scale; OCD, obsessive‐compulsive disorder; OR, odds ratio; SCAS, Spence Children's Anxiety Scale; VIF, variance inflation factor.

## DISCUSSION

In this study, we identified several key findings regarding the impact of the COVID‐19 pandemic on suicide‐related behaviors and associated clinical characteristics among elementary and junior high school students. First, the proportion of students with suicide‐related behaviors increased significantly after the pandemic, from 3.0% to 6.9% among elementary school students and from 15.5% to 21.2% among junior high school students. Second, anxiety symptoms, particularly social anxiety, panic, and fear of trauma, were more common among junior high school students with suicide‐related behaviors, whereas depressive symptoms did not differ consistently between the pre‐ and post‐COVID‐19 groups. Finally, antisocial behavior and behavioral and emotional disorders (ICD‐10 code F9) were more prevalent among elementary school students.

The first major finding was that the proportion of elementary and junior high school students presenting with suicide‐related behaviors at their initial psychiatric outpatient visit increased significantly after the spread of COVID‐19. Systematic reviews have reported a global rise in suicidal ideation, suicide attempts, and self‐injury among adolescents and young adults following the pandemic,^6^ which is consistent with our results. However, those studies did not examine detailed psychiatric symptoms. In contrast, our study focused specifically on elementary and junior high school students attending a psychiatric outpatient clinic, providing more precise clinical data. To address the absence of standardized methods for evaluating suicide‐related behaviors in prepubertal children,^26^ we combined diagnostic interviews with self‐administered depression and anxiety scales, conducted by psychiatrists trained in child psychiatry. This approach enabled comprehensive, developmentally appropriate assessments. Moreover, unlike demographic studies such as that of Goto et al., which analyzed suicide trends among Japanese youth aged 10**–**19 years without examining clinical characteristics,^10^ our study clarified developmental differences in suicide‐related behaviors and psychiatric symptoms. Another strength was the use of long‐term data spanning 2016 to 2022, which allowed robust comparisons before and after the pandemic.

The second major finding concerns depressive and anxiety symptoms. Analyses of these symptom scores were exploratory. Anxiety‐related symptoms, particularly social anxiety, panic, and fear of trauma, showed post‐pandemic increases, whereas depressive symptoms did not differ consistently between the pre‐ and post‐COVID‐19 groups. In the multivariate analysis of all junior high school students, DSRS scores were slightly lower in the post‐COVID‐19 group; however, no significant difference emerged when analyses were restricted to those with suicide‐related behaviors.

This finding contrasts with previous studies reporting global increases in both anxiety and depression^4^, ^11^ and may reflect differences in study populations and demographic factors. For example, Gracia‐Liso et al. investigated adolescents admitted to psychiatric emergency services following suicide attempts,^11^ whereas the present study included patients with suicide‐related behaviors at their first outpatient visit. Differences in patient acuity and referral context between emergency and outpatient samples may have influenced the pattern of symptom changes observed after the pandemic. Furthermore, these inconsistencies may also reflect ceiling effects within a clinically high‐risk sample that already exhibited elevated baseline symptom severity.

As for overall clinical characteristics, clear developmental contrasts were also evident. Elementary school students more frequently exhibited externalizing symptoms and F9 diagnoses, while junior high school students more often presented with internalizing symptoms, particularly anxiety. The internalizing–externalizing framework introduced by Achenbach et al.^27^ has been widely applied to classify psychiatric symptoms in children and adolescents and is closely linked to suicide‐related behaviors.^28^, ^29^ Externalizing symptoms such as rule‐breaking have been reported among preadolescents with suicide‐related behaviors,^30^ while internalizing symptoms, including anxiety and depression, are well‐established predictors in adolescents.^31^ Both domains reportedly increased among children and adolescents during the pandemic,^32^ consistent with our findings. Extending prior research, we demonstrated that externalizing symptoms increased significantly among elementary school students, while internalizing symptoms increased among junior high school students, highlighting developmental differences in symptom profiles associated with suicide‐related events.

Based on these results, changes in suicide‐related behaviors and psychiatric symptoms following the pandemic appear to differ by developmental stage. Elementary school students with suicide‐related behaviors demonstrated increased externalizing symptoms (e.g., antisocial behavior), while junior high school students showed more pronounced anxiety symptoms. Parental anxiety and stress during the pandemic may have disrupted children's emotional regulation and contributed to externalizing problems,^33^, ^34^ whereas in adolescents, such stress likely increased vulnerability to internalizing symptoms.^35^ Given that adolescence is marked by heightened sensitivity to social evaluation^36^ and that social connectedness buffers against internalizing symptoms,^37^ these findings enhance understanding of age‐specific suicide risks and psychiatric features, with implications for targeted prevention strategies.

This study has some limitations. First, it was a retrospective observational study at a single institution, which limits generalizability and precludes causal inference. Second, the dataset extended only through 2022; therefore, the ongoing effects of the pandemic could not be assessed. Suicides among children and adolescents in Japan have remained elevated even in 2024, and long‐term adverse effects not attributable solely to the acute phase of the pandemic have been reported both in Japan and internationally.^38^ These findings underscore the need for further long‐term follow‐up studies. Third, the significant differences observed in ADHD‐RS scores, diagnostic categories (F4, F5, F8, and F9), antisocial behavior, and anxiety subscales may have been influenced by multiple comparisons, referral pattern changes, or residual confounding. Notably, although the unadjusted proportion of females was higher in the post‐COVID group, multivariate analysis showed the opposite association (OR = 0.61), suggesting the presence of confounding factors. Fourth, assessments of suicide‐related behaviors were based on clinical diagnoses and self‐administered scales but also relied heavily on reports from patients and their families. Therefore, reporting and information biases cannot be excluded. For example, limited awareness or stigma may have contributed to underrecognition or underreporting of suicide‐related behaviors by family members,^39^ potentially leading to an underestimation of their prevalence.

In this study, we identified two key findings. First, the rate of suicide‐related behaviors increased significantly among both elementary and junior high school students after the pandemic. Second, anxiety symptoms were more common among junior high school students with suicide‐related behaviors, whereas no consistent differences in depressive symptoms were observed between the pre‐ and post‐COVID‐19 groups. Moreover, clinical characteristics varied by developmental stage: externalizing symptoms and conduct problems increased in elementary school students, whereas internalizing symptoms, particularly anxiety, were more prominent in junior high school students. These results underscore the importance of age‐appropriate suicide risk assessments. For elementary school students, evaluations should prioritize family environment and externalizing symptoms, while for junior high school students, assessments should focus on internalizing symptoms, especially anxiety. Developmentally tailored preventive approaches are particularly critical during global crises such as the COVID‐19 pandemic. Accurate assessment and early identification of high‐risk individuals remain essential for effective suicide prevention in child and adolescent psychiatry.

## CONCLUSION

We examined changes in suicide‐related behaviors and associated clinical characteristics among elementary and junior high school students before and after the COVID‐19 pandemic. Suicide‐related behaviors increased in both groups. Among elementary school students, externalizing symptoms such as antisocial behavior were more common, whereas among junior high school students, anxiety symptoms—particularly social anxiety and panic—were more frequent. No consistent changes in depressive symptoms were observed. These findings reflect characteristics specific to the study population, including regional and demographic factors. A nuanced understanding of these trends is essential to guide early intervention and tailored mental health support as part of suicide prevention efforts for children and adolescents.

## AUTHOR CONTRIBUTIONS

Masahide Usami, Mayuna Ichikawa, Miki Matsudo, Mutsumi Ohashi, Yui Higashino, Yusuke Kono, Haruna Matsudo, Yuki Nomura, Minjae Ma, Yuuki Sakoh, Maiko Odaka, Kotoe Itagaki, Keita Yamamoto, Momoka Takahashi, Yuta Yoshimura, Saori Inoue, Masahiro Ishida, Kumi Inazaki, and Yuki Mizumoto contributed to data acquisition. Masahiro Ishida and Yoshinori Sasaki analyzed and interpreted the data. Masahiro Ishida drafted the manuscript. All authors critically reviewed and revised the manuscript and approved the final version.

## CONFLICT OF INTEREST STATEMENT

The authors declare no conflicts of interest.

## ETHICS APPROVAL STATEMENT

This study was approved by the Ethics Committee of the National Center for Global Medical Research (approval numbers NCGM‐G‐003042‐08 and NCGM‐S‐004487‐03) and conducted in accordance with the Declaration of Helsinki.

## PATIENT CONSENT STATEMENT

In Japan, based on the “Ethical Guidelines for Medical and Health Research Involving Human Subjects,” personal information may be provided to third parties without explicit consent if individual consent is obtained and the individuals are given prior notice and the opportunity to refuse participation. In this study, prior notice was posted, and no written or verbal informed consent was obtained. The guidelines state, “It is not always necessary to obtain informed consent from research participants. However, for observational research using historical clinical records rather than human tissue samples, researchers must disclose information about the conduct of the research, including the purpose of the research.” Accordingly, details about the study's purpose, methods, analysis, and refusal options were posted in the hospital's outpatient clinic and on its website. During the study period, all data were anonymized to protect patient confidentiality.

## CLINICAL TRIAL REGISTRATION

Not applicable, as this was a retrospective observational study using medical records.

## Data Availability

The data that support the findings of this study are not publicly available due to ethical restrictions and the sensitive nature of clinical data.

## References

[1] Brooks SK , Webster RK , Smith LE , Woodland L , Wessely S , Greenberg N , et al. The psychological impact of quarantine and how to reduce it: rapid review of the evidence. Lancet. 2020;395:912–920. 10.1016/S0140-6736(20)30460-8 32112714 PMC7158942

[2] Duden GS , Gersdorf S , Stengler K . Global impact of the COVID‐19 pandemic on mental health services: a systematic review. J Psychiatr Res. 2022;154:354–377. 10.1016/j.jpsychires.2022.08.013 36055116 PMC9392550

[3] Saulle R , De Sario M , Bena A , Capra P , Culasso M , Davoli M , et al. School closures and mental health, wellbeing and health behaviours among children and adolescents during the second COVID‐19 wave: a systematic review of the literature. Epidemiol Prev. 2022;46:333–352. 10.19191/EP22.5-6.A542.089 36384255

[4] Racine N , McArthur BA , Cooke JE , Eirich R , Zhu J , Madigan S . Global prevalence of depressive and anxiety symptoms in children and adolescents during COVID‐19: a meta‐analysis. JAMA Pediatrics. 2021;175:1142–1150. 10.1001/jamapediatrics.2021.2482 34369987 PMC8353576

[5] Wolf K , Schmitz J . Scoping review: longitudinal effects of the COVID‐19 pandemic on child and adolescent mental health. Eur Child Adolesc Psychiatry. 2024;33:1257–1312. 10.1007/s00787-023-02206-8 37081139 PMC10119016

[6] Dubé JP , Smith MM , Sherry SB , Hewitt PL , Stewart SH . Suicide behaviors during the COVID‐19 pandemic: a meta‐analysis of 54 studies. Psychiatry Res. 2021;301:113998. 10.1016/j.psychres.2021.113998 34022657 PMC9225823

[7] Morisaki N , Hangai M , Goto R , et al Nationwide survey on children's mental health during the COVID‐19 pandemic [in Japanese]. J Jpn Pediatr Soc. 2023;127:182.

[8] Ministry of Health, Labor and Welfare . White paper on suicide prevention. Tokyo: Ministry of Health, Labor and Welfare; 2024.

[9] Ministry of Health, Lab or and Welfare . White paper on suicide prevention. Tokyo: Ministry of Health, Labor and Welfare; 2025.

[10] Goto R , Okubo Y , Skokauskas N . Reasons and trends in youth's suicide rates during the COVID‐19 pandemic. Lancet Reg Health West Pac. 2022;27:100567. 10.1016/j.lanwpc.2022.100567 35966624 PMC9366131

[11] Gracia‐Liso R , Portella MJ , Puntí‐Vidal J , Pujals‐Altés E , Torralbas‐Ortega J , Llorens M , et al. COVID‐19 pandemic has changed the psychiatric profile of adolescents attempting suicide: a cross‐sectional comparison. Int J Environ Res Public Health. 2023;20:2952. 10.3390/ijerph20042952 36833651 PMC9956974

[12] Kessler RC , Berglund P , Demler O , Jin R , Merikangas KR , Walters EE . Lifetime prevalence and age‐of‐onset distributions of DSM‐IV disorders in the national comorbidity survey replication. Arch Gen Psychiatry. 2005;62:593–602. 10.1001/archpsyc.62.6.593 15939837

[13] Houghton S , Kyron M , Hunter SC , Lawrence D , Hattie J , Carroll A , et al. Adolescents' longitudinal trajectories of mental health and loneliness: the impact of COVID‐19 school closures. J Adolesc. 2022;94:191–205. 10.1002/jad.12017 35353417 PMC9087620

[14] Sasaki Y , Sasaki S , Sunakawa H , Toguchi Y , Tanese S , Saito K , et al. Evaluating the daily life of child and adolescent psychiatric outpatients during temporary school closure over COVID‐19 pandemic: a single‐center case–control study in Japan. Glob Health Med. 2022;4:159–165. 10.35772/ghm.2022.01001 35855068 PMC9243403

[15] Ministry of Education, Culture, Sports, Science and Technology . Notice on temporary nationwide closure of elementary, junior high and high schools and special needs schools in response to COVID‐19. Tokyo: MEXT; 2020. Available from: https://www.mext.go.jp/en/mext_00006.html

[16] Posner K , Oquendo MA , Gould M , Stanley B , Davies M . Columbia Classification Algorithm of Suicide Assessment (C‐CASA): classification of suicidal events in the FDA's pediatric suicidal risk analysis of antidepressants. Am J Psychiatry. 2007;164:1035–1043. 10.1176/ajp.2007.164.7.1035 17606655 PMC3804920

[17] Birleson P . The validity of depressive disorder in childhood and the development of a self‐rating scale: a research report. J Child Psychol Psychiatry. 1981;22:73–88. 10.1111/j.1469-7610.1981.tb00533.x 7451588

[18] Murata T . Depression in school children: a study using the Birleson childhood depression scale. Saishin Seishin Igaku (Latest Psychiatry). 1996;1:131–138.

[19] Spence SH . A measure of anxiety symptoms among children. Behav Res Ther. 1998;36:545–566. 10.1016/s0005-7967(98)00034-5 9648330

[20] Kakihara M , Himachi M . A study on the relationship between depressive and anxiety symptoms in children. Fukuyama Univ Ment Health Couns Bull. 2012;6:127–134.

[21] Ishikawa S , Sato H , Sasagawa S . Anxiety disorder symptoms in Japanese children and adolescents. J Anxiety Disord. 2009;23:104–111. 18555658 10.1016/j.janxdis.2008.04.003

[22] DuPaul GJ , Power TJ , Anastopoulos AD , Reid R . ADHD Rating Scale‐IV: checklists, norms, and clinical interpretation. The Guilford Press; 1998.

[23] Takayanagi N , Yoshida S , Yasuda S , Adachi M , Kaneda‐Osato A , Tanaka M , et al. Psychometric properties of the Japanese ADHD‐RS in preschool children. Res Dev Disabil. 2016;55:268–278. 27164481 10.1016/j.ridd.2016.05.002

[24] Tani I , Okada R , Ohnishi M , Nakajima S , Tsujii M . Japanese version of home form of the ADHD‐RS: an evaluation of its reliability and validity. Res Dev Disabil. 2010;31:1426–1433. 10.1016/j.ridd.2010.06.016 20638822

[25] Kanda Y . Investigation of the freely available easy‐to‐use software “EZR” for medical statistics. Bone Marrow Transplant. 2013;48:452–458. 10.1038/bmt.2012.244 23208313 PMC3590441

[26] Ayer L , Ohana E , Ivanova MY , Frering HE , Achenbach TM , Althoff RR . Emotional and behavioral problem profiles of preteens with self‐injurious thoughts and behaviors: a multicultural study. J Am Acad Child Adolesc Psychiatry. 2024;63:931–942. 10.1016/j.jaac.2023.11.012 38280415 PMC11269522

[27] Achenbach TM , Ivanova MY , Rescorla LA , Turner LV , Althoff RR . Internalizing/externalizing problems: review and recommendations for clinical and research applications. J Am Acad Child Adolesc Psychiatry. 2016;55:647–656. 10.1016/j.jaac.2016.05.012 27453078

[28] Soto‐Sanz V , Castellví P , Piqueras JA , Rodríguez‐Marín J , Rodríguez‐Jiménez T , Miranda‐Mendizábal A , et al. Internalizing and externalizing symptoms and suicidal behaviour in young people: a systematic review and meta‐analysis of longitudinal studies. Acta Psychiatr Scand. 2019;140:5–19. 10.1111/acps.13036 30980525

[29] Commisso M , Temcheff C , Orri M , Poirier M , Lau M , Côté S , et al. Childhood externalizing, internalizing and comorbid problems: distinguishing young adults who think about suicide from those who attempt suicide. Psychol Med. 2023;53:1030–1037. 10.1017/S0033291721002464 34183077

[30] Ben‐Yehuda A , Aviram S , Govezensky J , Nitzan U , Levkovitz Y , Bloch Y . Suicidal behavior in minors—diagnostic differences between children and adolescents. J Dev Behav Pediatr. 2012;33:542–547. 10.1097/01.DBP.0000415830.85996.e6 22926661

[31] Piqueras JA , Soto‐Sanz V , Rodríguez‐Marín J , García‐Oliva C . What is the role of internalizing and externalizing symptoms in adolescent suicide behaviors. Int J Environ Res Public Health. 2019;16:2511. 10.3390/ijerph16142511 31337102 PMC6679016

[32] Levante A , Martis C , Bianco F , Castelli I , Petrocchi S , Lecciso F . Internalizing and externalizing symptoms in children during the COVID‐19 pandemic: a systematic mixed studies review. Front Psychol. 2023;14:1182309. 10.3389/fpsyg.2023.1182309 37397311 PMC10313408

[33] Dubois‐Comtois K , Suffren S , St‐Laurent D , Milot T , Lemelin JP . Child psychological functioning during the COVID‐19 lockdown: an ecological, family‐centered approach. J Dev Behav Pediatr. 2021;42:532–539. 10.1097/DBP.0000000000000935 34518496 PMC8432605

[34] Dollberg DG , Hanetz‐Gamliel K , Levy S . COVID‐19, child's behavior problems, and mother's anxiety and mentalization: a mediated moderation model. Curr Psychol. 2021;42:11733–11744. 10.1007/s12144-021-02476-y PMC8577641 34776719

[35] Liang Z , Mazzeschi C , Delvecchio E . The impact of parental stress on Italian adolescents' internalizing symptoms during the COVID‐19 pandemic: a longitudinal study. Int J Environ Res Public Health. 2021;18:8074. 10.3390/ijerph18158074 34360369 PMC8345594

[36] Rapee RM , Oar EL , Johnco CJ , Forbes MK , Fardouly J , Magson NR , et al. Adolescent development and risk for the onset of social‐emotional disorders: a review and conceptual model. Behav Res Ther. 2019;123:103501. 10.1016/j.brat.2019.103501 31733812

[37] Magson NR , Freeman JYA , Rapee RM , Richardson CE , Oar EL , Fardouly J . Risk and protective factors for prospective changes in adolescent mental health during the COVID‐19 pandemic. J Youth Adolesc. 2021;50:44–57. 10.1007/s10964-020-01332-9 33108542 PMC7590912

[38] Kiviruusu O , Ranta K , Lindgren M , Haravuori H , Silén Y , Therman S , et al. Mental health after the COVID‐19 pandemic among Finnish youth: a repeated, cross‐sectional, population‐based study. Lancet Psychiatry. 2024;11:451–460. 10.1016/S2215-0366(24)00108-1 38760112

[39] McGillivray L , Rheinberger D , Wang J , Burnett A , Torok M . Non‐disclosing youth: a cross sectional study to understand why young people do not disclose suicidal thoughts to their mental health professional. BMC Psychiatry. 2022;22:3. 10.1186/s12888-021-03636-x 34983460 PMC8728900

